# Responsiveness of different pain measures and recall periods in people undergoing surgery after a period of splinting for basal thumb joint osteoarthritis

**DOI:** 10.1186/s12874-022-01527-7

**Published:** 2022-02-05

**Authors:** Jenni Pajari, Jarkko Jokihaara, Eero Waris, Simo Taimela, Teppo L. N. Järvinen, Rachelle Buchbinder, Teemu Karjalainen

**Affiliations:** 1 Department of Surgery, Mikkeli Central Hospital, Mikkeli, Finland; 2 Tampere University and Tampere University Hospital, Tampere, Finland; 3 Department of Surgery, Helsinki University and Helsinki University Hospital, Helsinki, Finland; 4 Finnish Centre for Evidence-Based Orthopaedics (FICEBO), Department of Orthopaedics and Traumatology, University of Helsinki and Helsinki University Hospital, Topeliuksenkatu 5, HUS, 00029 Helsinki, Finland; 5 Monash Department of Clinical Epidemiology, Cabrini Institute, Melbourne, Australia; 6 Department of Epidemiology and Preventive Medicine, School of Public Health & Preventive Medicine, Monash University, Melbourne, Australia; 7 Cochrane Musculoskeletal Group, Clinical Trials Unit, Warwick Medical School, Warwick University, Melbourne, Australia; 8 Department of Surgery, Hospital Nova Central Finland, Hoitajantie 3, 40620 Jyvaskyla, Finland; 9 Monash Department of Clinical Epidemiology, Cabrini Health and Department of Epidemiology and Preventive Medicine, School of Public Health & Preventive Medicine, Monash University, Malvern, Australia

**Keywords:** Pain, Osteoarthritis, Thumb, Recall time

## Abstract

**Background:**

Basal thumb joint osteoarthritis (OA) is a common painful condition of the hand often treated surgically if non-operative care does not provide sufficient pain relief. Many instruments are available to measure pain for this condition including single item and multidimensional measures. To inform our choice of instrument for the purpose of evaluating the value of surgery for people with thumb OA, the aim of this study was to compare the longitudinal validity and signal to noise ratio of a single item numeric rating scale (NRS) for pain and the Patient-rated Wrist and Hand Evaluation (PRWHE) pain subscale, and to assess if recall period affects longitudinal validity of the NRS pain and reported pain levels.

**Methods:**

We invited 52 patients referred for surgical treatment of basal thumb joint OA to participate in this study. All wore a splint for six weeks followed by surgery. Pain during the past day, week, and month and the PRWHE were collected at baseline, operation day, and 3, 6, 9 and 12 months after surgery. Responsiveness was assessed with two methods: 1) using participant-reported global improvement and PRWHE function subscale as external anchors (longitudinal validity) and 2) comparing Standardized Response Means (SRM).

**Results:**

The Spearman’s ρ between PRWHE pain and participant-reported global improvement was better (0.71) compared with NRS past day (0.55), past week (0.62), or past month (0.59). Similar findings were found with PRWHE function as anchor (Pearson’s r for PRWHE pain 0.78; NRS past day 0.68; past week 0.73; past month 0.69). The SRM of PRWHE pain subscale (2.8) and NRS past week (2.9) outperformed pain past day (2.3) and month (2.4). Mean pain was 0.3 points (on a 0 to 10 scale) worse during past week when compared with past day and 0.3 worse during past month than during past week.

**Conclusions:**

All studied pain measures captured the change in pain over time. For clinical trials, we recommend PRWHE pain subscale or NRS past week due to their better signal noise ratio.

**Trial registration:**

Retrospectively registered.

## Background

Basal thumb joint osteoarthritis is one of the most common painful conditions of the hand typically affecting the quality of life of middle aged and elderly people [1–5]. Anti-inflammatory drugs, intra-articular glucocorticoid injections and various orthoses can be used for pain relief. These treatments, however, may not mitigate symptoms sufficiently, and in those cases surgery – most commonly removal of the trapezoid bone (trapeziectomy) – is used to relieve pain. Although observational data suggests this procedure relieves pain, there are no studies that compare surgery with non-operative treatment [6].

Since pain is a subjective experience, patient-reported outcomes are used to assess the effect of interventions [7]. A systematic review assessing the outcomes used in basal thumb joint OA identified 101 different outcome measures, most frequently measuring pain and function domains [8]. Pain can be evaluated with various measures. A single item Visual Analog Scale (VAS) and Numeric Rating Scale (NRS) are common methods for measuring OA thumb pain. However, single item instruments can only measure one dimension of pain, such as its intensity, and they cannot capture different aspects of the pain experience such as its fluctuation, frequency and quality [9]. Another potential issue with pain questions is the recall period, which is often defined as current pain, pain during the past 24 h, or past week.

The difference between recalled pain and real time mean pain measurement is called recall bias [10]. Recall bias is usually greater with longer recall periods, such as a week or a month, when compared with the past day [11]. Furthermore, in musculoskeletal pain, studies suggest that the recall period of the question may influence the reported pain levels [11, 12] but it is not clear if this is a universal phenomenon. It is also unclear if the recall period (and related recall bias) can impact the longitudinal validity of the measure, i.e. how well the measure captures the change in the underlying construct.

The Patient-Rated Wrist and Hand Evaluation (PRWHE) is a hand-specific instrument which includes a pain subscale comprising five items; four measure pain intensity (at rest, when doing a task with a repeated wrist movement, when lifting a heavy object and when it is at its worst), and one measures pain frequency. In theory, the benefit of multiple items is that an overall pain score better captures all important aspects of a person’s pain. Furthermore, use of multiple items could improve the signal to noise ratio as the random errors of different responses cancel each other to some extent when the total score is summed from several items. These properties could improve the ability to measure the change. However, the evidence informing whether multi-item PRWHE is better choice for clinical trials compared with single item NRS is limited.

The aim of this study was to determine 1) if change in PRWHE pain subscale correlates better than a single item pain NRS with participant-reported global improvement or hand function following treatment (i.e. compare longitudinal validity of NRS and PRWHE pain); 2) if the PRWHE pain subscale has a better signal to noise ratio than the single item pain NRS; and 3) if the recall period affects the longitudinal validity of single item NRS or 4) self-reported mean pain levels of a single item NRS pain in an observational cohort of participants who underwent sequential splinting then surgical intervention for basal thumb joint OA.

## Methods

### Study design and setting

The study was a multi-center prospective observational cohort study. We recruited participants from one secondary and two tertiary referral centers between August 2017 and November 2018. All participants had been referred for operative treatment for symptomatic basal thumb joint OA that had not responded to non-operative treatment. Helsinki University Hospital Institutional Review Board approved the study protocol before commencement of the study. All methods were carried out in accordance with relevant guidelines and regulations.

### Study hypotheses

In people being treated for basal thumb joint OA, our null-hypotheses were:

1. The longitudinal validity of the PRWHE pain subscale and a single item pain NRS are not significantly different (measured as the correlation between target instrument and participant-reported global improvement or hand function improvement)
2. The signal to noise ratio (expressed as a standardized response mean, SRM) of the PRWHE pain subscale is not significantly different from the NRS.
3. The length of the recall period does not affect the longitudinal validity of the pain NRS (correlation between improvement in pain and participant-reported global improvement or hand function improvement)
4. Difference in the length of the recall period does not affect the reported level of pain.

### Participants

All people referred for basal thumb joint OA were screened by a board-certified hand surgeon. If they were dissatisfied with the pain and/or function of the hand, and accepted the risks related to surgery, standard surgical care (trapeziectomy) was offered and they were informed about the study. Recruitment occurred after the decision to operate was made.

Inclusion criteria

- Basal thumb (carpometacarpal) joint OA (Eaton-Littler grades 2-4)
- > 45 years of age
- Scheduled for trapeziectomy

Exclusion criteria

- Systemic inflammatory disease
- Neurological disease that weakens upper arm muscles
- Any operation of the same hand during the past 6 months
- Bilateral operation planned
- Zig zag deformity (> 45 degrees of thumb metacarpophalangeal (MP) joint hyperextension in rest)

### Interventions

After recruitment, all participants were given an Actimove® Rhizo Forte splint (Essity AB, Stockholm, Sweden), and they were instructed to use it continuously for six weeks. After splinting period, all participants underwent a simple trapeziectomy. The trapezoid bone was exposed using a volar approach and removed in a piecemeal fashion. The space between first metacarpal and scaphoid was carefully examined for any bone remnants before the remaining joint capsule was closed. We did not use any interposition or suspensionplasty. After skin closure, the thumb was immobilized with a cast in palmar abduction/opposition for 3 weeks. At 3 weeks, the cast was removed and participants were provided with a post-operative exercise protocol that they could perform at home daily. They were also instructed to use the orthosis in daily activities as needed for the next three weeks and after that only if needed to control the pain.

### Outcomes

The baseline characteristics were recorded after the participants had signed the informed consent. Questionnaires were sent by mail to all participants. The outcome measures were collected at baseline, after 6 weeks of splinting, and then 3 months, 6 months, 9 months and 12 months post-surgery.

Pain was measured by two instruments:

1. a single item 11-point NRS where 0 = no pain and 10 = worst possible pain. The same question was posed three times with different recall periods: “*How would you describe the pain in your thumb during the past day / during the past week / during the past month while doing daily activities, work and/or hobbies?”*. The intraclass correlation coefficient (ICC) in NRS with knee OA patients is 0.95, whereas standard error of measurement (SEM) is 0.48 and minimum detectable difference (MDD) 1.33 [13]. The minimal important difference (MID) is 2.0 with chronic musculoskeletal patients [14].

2) the PRWHE pain subscale which comprises five items as described above. The four items assessing pain intensity are measured on an 11-point NRS scale where 0 = no pain and 10 = worst possible pain. The recall period is one week. The fifth item assessing pain frequency is measured on an 11-point NRS scale where 0 = never to 10 = always. The pain subscale is the sum of the five items and ranges from 0 (best) to 50 (worst). PRWHE is the same instrument as the PRWE [15] but the word ‘wrist’ is replaced by ‘hand’. PRWE has been validated in Finnish [16].

All pain items were in the same order in the questionnaires: day, week and month.

The anchor measures in the assessment of longitudinal validity were

1. participant-reported global improvement measured by a 5-point Likert scale (*much better; little better; no change; little worse; much worse*).
2. the PRWHE function subscale which comprises 10 items rating the difficulty in doing specific tasks during the past week on a NRS scale from 0 = not difficult to 10 = unable to do. The PRWHE function subscale is the sum of the 10 items and ranges from 0 (no disability) to 100 (worst disability). Systematic review found an ICC of 0.85 for PRWHE [17], SEM was 2 points with various hand surgery patients, [18] MDD was 11 points, and minimal clinically important difference (MCID) was 11.5 points in patients with distal radius fracture [19].

### Statistical analyses

Change scores were calculated by subtracting baseline score from the follow-up scores. We changed the direction of the pain scales so that a positive value corresponds with improvement in pain. We used measurements from all time points for the longitudinal validity assessment (50 target measure–anchor measure data pairs at five follow-up points = 250 data pairs), and also for the comparison of different recall periods of the single item NRS (6 time points * 3 question * 50 participants = 900 observations).

To compare the longitudinal validity of the three pain NRS recall periods (past day, week, month) and the PRWHE pain subscale, we calculated Spearman correlations between the target measures and participant-reported global improvement; and Pearson correlations with the target measures and change in PRWHE function subscale. Confidence intervals for the correlations were derived by bootstrapping 1000 samples. We then compared these correlations between measures using the method described by Steiger [20].

To assess the signal/noise to ratio, SRMs were calculated as change in score / SD of change for all pain outcomes using data from baseline and at 12 months after surgery. We compared the SRMs of the single item pain NRS scores and the PRWHE pain subscale scores using a “modified Jack-knife test” similar to Angst et al. [21].

We estimated the difference in the reported pain in different recall periods using a linear mixed model. Participant was entered as random factor and the question type (pain during past day, week, or month) and time points as fixed factors. We also fitted another model assessing if the time from recruitment modified the effect (time point*question interaction). Estimated marginal means from the mixed model were used as mean values. In all analyses, *p*-values less than 0.05 were considered statistically significant.

## Results

### Baseline characteristics

In total, we recruited 52 participants. Two participants did not attend any of the follow-up visits and thus complete data were available for 50 (96%) participants at all follow-up points. Their demographic data and outcome variables at baseline are shown in Table 1. The study population was predominantly female (75%). Most of the participants (80%) had Eaton-Littler grade 3 or 4 basal thumb joint OA.

**Table 1:** Descriptive characteristics and baseline variables for the included participants (*N* = 50)

	N (%)
**Female**	39 (75)
**Handedness**	
Right	43 (83)
Left	6 (12)
Ambidextrous	3 (5.8)
**Affected side**	
Dominant	19 (37)
Non-dominant	27 (52)
Bilateral^a^	6 (12)
**Smoker**	11 (21)
**Eaton-Littler classification**	
Stage 2	9 (17)
Stage 3	20 (39)
Stage 4	22 (42)
	Mean (SD)
**Age, years**	62 (7.6)
**BMI, kg/m**^2^	28 (4.4)
**Pain NRS, past day (0 to 10)**^b^	6.5 (1.7)
**Pain NRS, past week (0 to 10)** ^b^	6.9 (1.6)
**Pain NRS, past month (0 to 10)** ^b^	6.9 (1.6)
**PRWHE pain subscale (0 to 50)** ^b^	35 (7.2)

^a^ Surgery was only performed in patients with bilateral symptoms for the most symptomatic hand

^b^ Higher score indicates worse pain

### Responsiveness

The PRWHE pain subscale displayed slightly better longitudinal validity (correlation with both patient-reported global improvement and change in PRWHE function) compared with any of the single item pain NRSs (Table 2). Recall period for the single item NRS did not influence its longitudinal validity (*p* > 0.05 for all comparisons). The SRM of the PRWHE pain scale (2.8) was similar to the single item pain NRS with a recall period of during the past week (2.9), but better than the NRS with recall periods of during the past day (2.3) and during the past month (2.4) (Table 3).

**Table 2:** Longitudinal validity (correlations between different pain measures and anchor measures)

**A) participant-reported global improvement**	**Spearman’s rho**	**95% CI**	*p***-value compared to** **PRWHE pain**^a^
**PRWHE pain subscale**	0.71	0.64 to 0.78	ref
**Pain NRS, past day**	0.55	0.45 to 0.64	*p* < 0.001
**Pain NRS, past week**	0.62	0.54 to 0.70	*p* = 0.004
**Pain NRS, past month**	0.59	0.50 to 0.68	*p* < 0.001
**B) PRWHE function subscale**	**Pearson’s correlation coefficient**	**95% CI**	*p***-value compared to** **PRWHE pain**
**PRWHE pain subscale**	0.78	0.73 to 0.83	ref
**Pain NRS, past day**	0.68	0.60 to 0.75	*p* < 0.001
**Pain NRS, past week**	0.73	0.66 to 0.78	*p* = 0.017
**Pain NRS, past month**	0.69	0.63 to 0.79	*p* = 0.020

^a^ *P*-value from a test comparing the Spearman’s rho or Pearson’s correlation co-efficient between the measures [20]

**Table 3:** Standardized response means, SRM

Outcome	SRM	95% CI	*p*-value compared to PRWHE pain
**PRWHE pain subscale**	2.8	2.6 to 3.1	ref.
**Pain NRS, past day**	2.3	2.1 to 2.6	*p* = 0.001
**Pain NRS, past week**	2.9	2.7 to 3.2	*p* = 0.56
**Pain NRS, past month**	2.4	2.1 to 2.7	*p* = 0.001

### Effect of NRS recall period

**Fig. 1:**
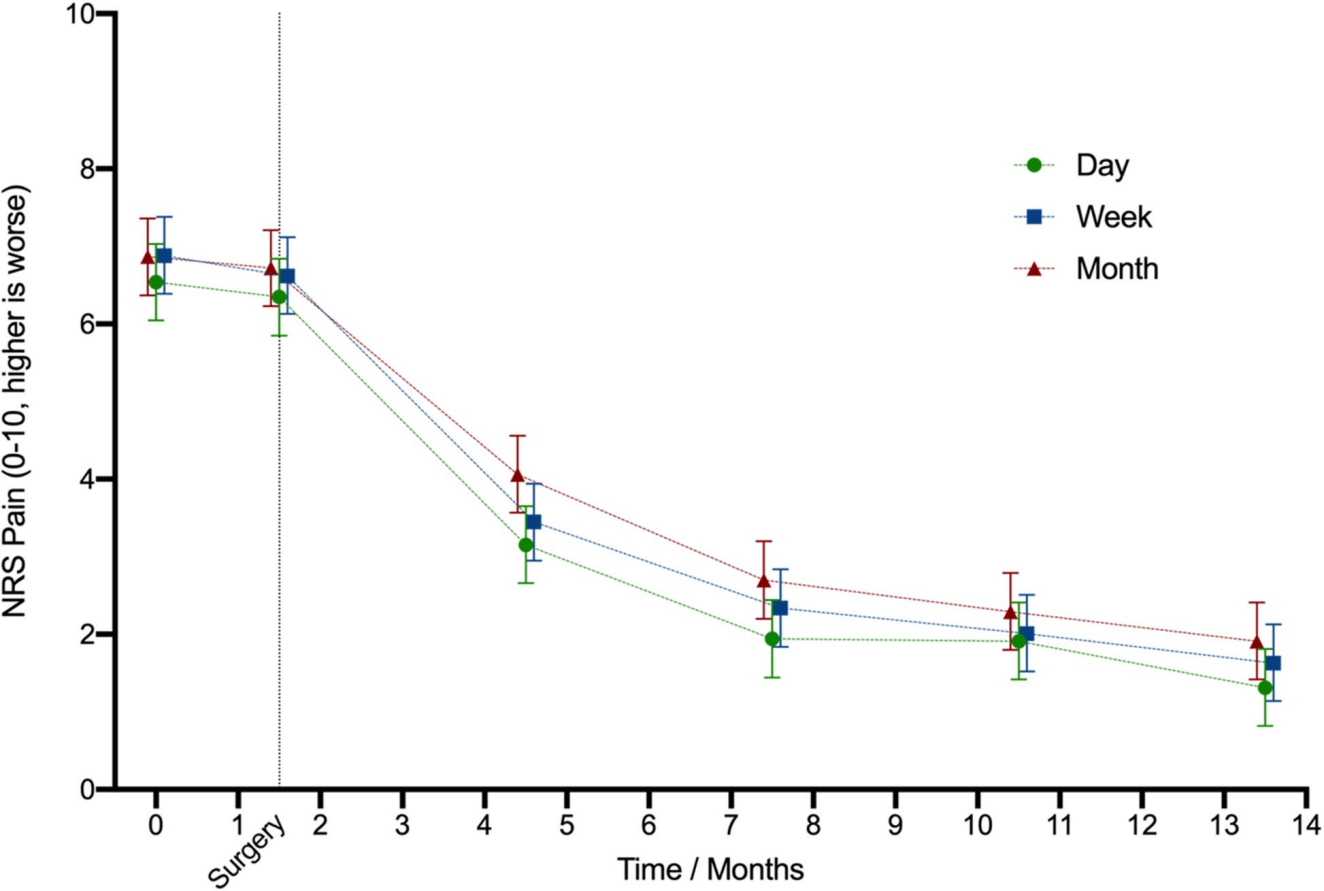
Pain at each timepoint for each single item pain NRS recall period

Pain did not change during the 6-week splinting period, but it decreased steadily after surgery. (Fig. 1). The duration of the recall period affected pain NRS values significantly; the longer the recall period, the higher the reported pain (Table 4). The 0.3-point difference between day and week and between week and month was consistent across all time points (Fig. 1). The duration of follow-up did not modify this difference (time*question interaction; day versus week *p* = 0.8; day versus month p = 0.8).

**Table 4:** The mean difference across all time points for different recall periods

	Estimate	95% CI	*p*
**Pain NRS, past day**	3.53^a^	1.7 to 5.4 (ref.)	ref.
**Pain NRS, past week**	0.29	0.06 to 0.52	0.011
**Pain NRS, past month**	0.56	0.33 to 0.78	< 0.001

^a^ Mean value across all time points (reference); other estimates indicate the difference compared to reference (past day

## Discussion

The PRWHE pain subscale was slightly more sensitive to change, i.e., it had better longitudinal validity compared with a single item pain NRS, but all measures seemed to capture change in pain well. On the other hand, PRWHE pain subscale and NRS past week had best signal to noise ratio translating into greater precision of treatment estimates in clinical trials making them best options for research use. Longer recall periods resulted in small increments in reported pain levels with NRS, but the recall period did not impact the longitudinal validity of the measure.

Sensitivity to detect change in a measured construct can be assessed by 1) comparing change to an external anchor (‘external responsiveness’ or longitudinal validity), i.e. measuring the extent to which change relates to a reference measure of health status; or 2) calculating varying signal to noise ratios (‘internal responsiveness’), that characterize the ability to measure change in relation to the variability of the measurement (SRM in this study) [22]. The PRWHE pain subscale seems to outperform single NRS pain items in external responsiveness and is better than pain during past day and month in internal responsiveness while the benefit of NRS is acceptable longitudinal validity with less response burden.

Our finding agrees with a previous study comparing the Multidimensional Pain Inventory (MPI) with a single item pain NRS in which the MPI showed better responsiveness [21]. On the other hand, a more comprehensive evaluation does not automatically result in better responsiveness: the McGill pain questionnaire, which has 49 items including several items for pain quality and intensity, was less responsive when compared with a single pain VAS in people with low back pain [23]. The PRWHE pain subscale measures pain intensity (4 items) and pain frequency (1 item), and it seems to capture important aspects of thumb pain more comprehensively than a single item pain NRS.

The data do not shed light on the mechanisms regarding the recall period of pain, but the results are in line with other studies assessing recall bias, which have also found lower pain values with shorter recall periods [11, 12]. Evidence also suggests, that as the recall period grows longer, the reported pain values tend to correlate less with the real time pain measurements, indicative of recall bias [11]. This bias likely relates to how people construct the past in their mind – recalled pain seems to correlate best with the worst pain experienced and pain at the end of the period rather than the average pain over the whole time period [24, 25]. Arthritic pain intensity typically fluctuates and it is more likely that a person experiences peaks in longer recall periods compared with shorter periods. Thus, it is plausible that participants reported, on average, slightly higher pain during the past week and past month when compared with past day.

The main limitation of this study is external validity. All participants had been referred to a hand surgical center due to persisting painful basal thumb joint OA and it is unclear if the results can be directly applied to other hand joints or patients with less severe symptoms. However, as pain recall bias is a psychological phenomenon, we don’t see any reason why the results would markedly differ in other OA joints, but further investigation is warranted. We also did not assess how an overall pain value (a single item pain NRS without a specific recall period) performs, and the anchor questions can also be subject to recall bias [26]. Also, the order of NRS pain questions was not randomized and this could impact their relative values.

We also did not record real time pain in order to assess which measurement is least biased from real time values. There is no gold standard for pain measurement and it is unclear if real time pain is more important compared with recalled pain but people seem to put more weight on the recalled pain than currently experienced pain in decision making [17].

## Conclusions

Of the tested measures, we recommend PRWHE pain subscale or NRS past week for clinical trials. The slightly lower longitudinal validity of NRS past week can be weighed against the benefit of having lower response burden, at least in clinical practice when a multiple item questionnaire is not feasible. Since the recall period does not seem to impact the longitudinal validity of the measurement, it is not an important issue within a trial, but the difference in the absolute level caused by recall period may be factored in when absolute values are compared across trials with different pain recall periods.

## Data Availability

The datasets used and/or analyzed during the current study are available from the corresponding author on reasonable request.

## Abbreviations

NRS pain: Numeric pain rating scale
PRWHE: Patient-rated Wrist and Hand Evaluation
OA: Osteoarthritis
SRM: Standardized Response Means
VAS: Visual Analog Scale
MP: Metacarpophalangeal
PRWE: Patient-Rated Wrist Evaluation
MPI: Multidimensional Pain Inventory
ICC: Intraclass Correlation Coefficient
SEM: Standard error of measurement
MDD: minimum detectable difference
MID: The minimal important difference
MCID: minimal clinically important difference
